# An Exploratory Study of Adult Baby-Diaper Lovers’ Characteristics in an Italian Online Sample

**DOI:** 10.3390/ijerph17041371

**Published:** 2020-02-20

**Authors:** Antonietta Lasala, Francesco Paparo, Vincenzo Paolo Senese, Raffaella Perrella

**Affiliations:** Department of Psychology, University of Campania “Luigi Vanvitelli”, 81100 Caserta, Italy; lasala.antonietta@yahoo.com (A.L.); francesco_paparo@yahoo.it (F.P.); vincenzopaolo.senese@unicampania.it (V.P.S.)

**Keywords:** adult baby, diaper lovers, paraphilia, paraphilic infantilism, fetishism

## Abstract

Background: Knowledge of the Adult Baby-Diaper Lovers (ABDL) phenomena is quite recent and there are, of yet, few studies on this phenomenon. Aim: This study was conceived to investigate the functions of ABDL behaviours and the characteristics of ABDL in an online Italian community sample. We hypothesized that ABDL phenomena were associated with general psychological maladjustment and with an experience of parental rejection during childhood. It was also assumed that there would be differences in ABDL profiles based on the age of appearance of their first Adult Baby-Diaper Lover (ABDL) fantasies. Method: An internet-based study was conducted and it involved 38 adults aged between 18 and 74 years (*M* = 34.95; *SD* = 12.25). Participants were first given an ad hoc questionnaire, which was devised to obtain information about the anamnestic variables related to ABDL. Then, the participants filled out the Cognitive Behaviour Assessment 2.0 battery to obtain anamnestic information regarding their psychological, medical, and personal history and to evaluate primary psychological dimensions in clinical practice. Finally, they filled out the Adult Parental Acceptance–Rejection Questionnaire, to evaluate their recollections of parental perceived rejection, and the Personality Assessment Questionnaire, to evaluate the primary psychological aspects related to parental rejection. Results: The data indicated that adults with ABDL showed the presence of anxious traits and recollections of parental rejection during childhood. Moreover, associations were observed between current or previous ABDL phenomena enuresis and negative mood states. Conclusion: Specific kinds of parental modes, anxiety traits, and enuresis seem to be the source of ABDL interests. Moreover, ADBL behaviours seem to assume different functions and meanings.

## 1. Introduction

Typical and atypical sexuality is difficult to examine due to the intimate and variable nature of the study. Furthermore, the notion of sexual deviation changes with regard to different historical ages and, in its definition, is influenced by socio-cultural variables [1]. DSM-5 defines paraphilia as: “any powerful and persistent sexual interest other than sexual interest in copulative or pre-copulative behaviour with phenotypically normal, consenting adult human partners” [2] (pp 794). In recent years, in the paraphilic field, a peculiar phenomenon has been described, called Adult Baby/Diaper Lovers (ABDL), which concerns persons who act a voluntary regression to a previous age and/or wear a diaper for psychological reasons [3]. 

The first documented cases of ABDL are dated to 1964, however, to date, there are still few studies that have described this phenomenon. Tuchman and Lachman [4] described a case of a 29-year-old male who regularly wore diapers and masturbated when he wore them. In 1967, Diniello [5] referred a case of man who had orgasms when he wore diapers, and he adopted other infant behaviours, such as eating baby food or drinking from a baby bottle. Benthell [6] reported a case of a man with brain damage for whom wearing little girl clothes and the diaper was necessary to have an erection and sexual gratification. Evciment and Gratz [7] described a 57-year-old male case who was hospitalized because he was affected by hallucinations and had declared to have a desire to be 3-years-old children and to be nurtured by his wife as if she were his mother. In this case, sexual fantasies were not present, and the treatment and resolution of hallucinations did not lead to the dissolution of ABDL fantasies. 

Kise and Nguyen [8] reported a case where ABDL interests were included in a gender dysphoria framework. The man required psychological counselling because of gender dysphoria but he did not perceive ABDL interests as a distressing factor. Cernowsky and Bureau [9] reported a case of a woman affected by depressive symptoms and ABDL interests who was asking for help only for the former symptoms, not for the latter. Banbury, Lusher, Lewis, and Turner [10] described a case of two adults who practiced ABDL and had a drug addiction, showing that in both cases, either the addiction and the ABDL practices could be considered an auto-medication practices carried out to face sexual abuses during childhood. In both cases, only the drug addiction was perceived as distressing or as a dangerous problem. Caldwell [11] referred to a man who masturbated while wearing a diaper and asked his wife that a diaper became a component of their sexual life. 

More recently, Zamboni et al. [12,13,14,15,16], to describe the more general characteristics of the ABDL population, conducted a study using a large sample of ABDL, by means of standardized measures. In particular, the authors investigated the ABDL features in a sample composed of 1934 members of the online ABDL community. The data showed that ABDL phenomena were diffuse mostly among males. Male members identified their own ABDL interests before females, and they focused on the sexual dimensions of ABDL practices. Both males and females perceived the component of being dominated as the primary pleasant aspect of the ABDL behaviour [12]. About half of the sample were in a romantic relationship. In some cases, the ABDL desires were satisfied with a romantic partner, but about 50% of partners disapproved of ABDL behaviours [13]. The majority of the sample did not experience distress for the ABDL behaviours, but when present, the distress was related to other factors, such as the partner’s knowledge of ABDL interests, the fear of being discovered or being misunderstood, or the feeling of shame for family members [14].

In summary, the analysis of the literature available so far shows that behaviouristic patterns of ABDL are variable. Indeed, for some persons ABDL behaviours have a sexual aim [4,5,6,11], whereas for other persons ABDL behaviours do not have a sexual motivation [7]. Hawkinson and Zamboni [12], in line with other authors [1,7,11], have hypothesized the existence of at least two different subgroups of ABDL. Indeed, in their study [12] they observed that the link between infant regression and diaper fetishism were not always present. For example, some persons that were more focused on the regressive dimension declared that they perceived sexual arousal for other childhood objects as well. This interest did not fit the necessary criteria for a fetishistic disorder. For this reason, the authors hypothesized that interests in infant regression could have different meanings and functions in these two subgroups. 

In the first group, ABDL behaviours seem to have a sexual nature and this condition has been named as “Diaperism” or “Diaper Lovers” (DL) [3]. In this group, some individuals are dominated by a fetishist interest in the diaper [9,11,12,17]; other individuals are excited by stool and urine release in a diaper. In this latter cases, a qualitative analysis made by Zamboni [15] showed that ABDL simulated excrementary functions with food or water to feel excited due to the dirty diaper’s contact with their genitals; other individuals can focus on diaper tissue and the tactile feel for diaper touch on the genitals [15]; whereas other individuals use ABDL behaviours as a part of a bondage discipline sadism and masochism (BDSM) sexual practice. In this case, sexual arousal concerns the submission and humiliation that are inside the sado-masochistic relationships [3,9,15]. For example, Zamboni and Madero [18] made an analysis of asexual individuals in the online ABDL community, confirming the hypothesis that ABDL, like BDSM, could represent a way to decrease anxiety and to sexually satisfy a partner. 

In the second group, ABDL behaviours seem to have a mainly regressive nature or function. This condition is known as “Paraphilic Infantilism”, “Autonephiofilia” [19], “Adult Baby Syndrome” (ABs) [20], or “Adult Baby” (AB). Members of this group feel a wish to be a baby, adopting typical infant behaviour, such as wearing diapers, drinking from a baby bottle, playing with baby toys. These behaviours are likely aimed to encourage identification with a baby and to simplify infant regression [12,16,18]. Considering that for AB individuals neither research of sexual arousal nor sexual pleasure is a priority, it is not possible to consider this practice as a paraphilic interest or behaviour [10]. 

In this case, AB behaviours can be in response to different functions for different people [12]. For example, for some individuals, AB behaviour could have an interpersonal nature and result from anxious attachment with the main caregiver. For example, the infantile regression can be inserted in a role-play, defined by Lewis [21] “Ageplay”, with the presence of a partner in a “mommy” or “daddy” role. Zamboni [13,16] showed a greater involvement of both a romantic partner and other partners in AB practice among persons more focused on enjoyment role-playing and with higher levels of anxious attachment. Zamboni [16] and Zamboni and Madero [18] hypothesized that for these persons, AB behaviour was a way to create social, romantic and/or affective links, decreasing simultaneously interpersonal anxiety. For other individuals, AB behaviours could be a way to relax, to avoid negative mood state or daily responsibility, even to become a lifestyle [8,10,11]. For other persons, instead, AB practices could reveal the attempt to re-live and change their own childhood [22] and to accomplish love and acceptance not felt during childhood [12]. Finally, individuals are defined as ABDL when no specific classification about the motivation of the practices has been done, or when they present both infant regression and sexual connotation [3,12].

Given the variability of ABDL manifestations, a specific cause for the ABDL behaviours cannot be identified. In the literature, different hypotheses have been proposed for the genesis of ABDL interests. According to some scholars [3,8,10], ABDL practices are related to traumatic developmental experiences. In this perspective, ABDL interests are considered as a way to elaborate psychological conflict consequent to traumatic experiences from childhood, such as sexual or physical abuse or experiences of victimization. 

For other authors, ABDL practices and fantasies are associated with a negative or inadequate parental relationship [16,22]. Supporting this perspective would be the extensive literature that highlights how an inadequate relationship with caregivers can lead to the development of atypical or paraphilic sexual desire [23]. Data confirmed the association between ABDL practices and the quality of the early parental relationship, showing that ABDL behaviours were more frequent in adults with an anxious attachment or negative parental relationships [12,16]. Moreover, Zamboni [16] found a positive correlation between AB role-play and being raised only by a mother. 

Other scholars associate the erotic fixation and infant regression observed in the ABDL practices to the presence of incontinence [9,11]. For example, Cernowsky and Bureau [9], according to a behaviouristic perspective, have hypothesized that incontinence episodes lived during childhood were associated with negative parental reactions, that these parental behaviours generated negative feelings for children, and that wearing diapers decreased the negative arousal, determining safety and protection feelings, thus reinforcing the association between ABDL behaviours and a positive state. A similar hypothesis was formulated by Zamboni [15], who observed that some ABDL participants reported that their practices were related to their incontinence. 

Based on the statements of some ABDL individuals, other scholars have hypothesized that ABDL behaviours can be considered the expression of more general dysfunctional coping strategies [10,11,22] carried out to avoid negative mood states, such as sadness and frustration, to decrease anxiety or to escape from daily responsibilities. This interpretation is also confirmed by the data observed by Hawkinson and Zamboni [12] that showed that, in males, the levels of anxiety or a negative mood state were positively associated with the frequency of AB role-play not with DL interest. Finally, according to some scholars [7,9,11] ABDL manifestations could reflect a behavioural spectrum, from paraphilic interest to obsessive-compulsive behaviours. In this perspective, ABDL could be a sub-clinical form of obsessive-compulsive disorder. 

Starting from the above-mentioned considerations, the present study is designed to highlight the existence and the diffusion of ABDL phenomena in Italy. In fact, given that ABDL phenomena are still an under investigated field and given the absence of previous research on ABDL in the Italian context, our aim was to provide descriptive information on both ABDL phenomena and persons who have this behaviour. In particular, we have assessed the presence of the main elements associated with ABDL in the literature in a specific Italian population: persons who use ABDL online communities to express and share their own ABDL interests. 

Moreover, as only one study showed an association between a negative or inadequate parental relationship and the appearance of ABDL fantasies [9], a second object of this study was to investigate if the adults with ABDL showed more negative recollections of parental acceptance–rejection compared with the general Italian population. The innovative aspect of our study consists of investigating, in a systematic way, the association between the degree of parental acceptance/rejection, both maternal and paternal, and ABDL practices. In particular, we hypothesized that ABDL interests are positively associated with the degree of experienced rejection from both parental figures. In this perspective, ABDL phenomena could be considered one of the possible consequences of perceived parental rejection during childhood [24,25,26]. 

Moreover, linked to this latter hypothesis, given that the literature (see Interpersonal Acceptance Rejection Theory, IPAR Theory, [26]) has widely confirmed the link between childhood rejection experiences and psychological adjustment, a further objective of this study was to assess whether the presence of ABDL fantasies were associated with a more negative psychological adjustment in general, when compared with the overall Italian population. Furthermore, given that, in the literature, ABDL phenomena were present in psychopathological disorders, such as depression [9] and obsessive-compulsive disorder [11], we wanted to obtain an evaluation of the main clinical problems most frequently encountered in clinical practice. 

In particular, on the one hand we wanted to exclude underlying psychopathological conditions causing ABDL phenomena, such as the presence of obsessions that lead to compulsive behaviours, such as wearing a diaper. On the other hand, we wanted to have a clarified framework, both on the associations between ABDL phenomena and on other psychological issues, such as depressive mood states, specific phobias, or stress and its effect on physical states. Finally, given that an extreme variability of ABDL phenomena have been observed, we have hypothesized that there could be differences in ABDL profiles based on the age of appearance of the first ABDL-related fantasies. Indeed, given that it has been observed that some adults reported the appearance of fantasies already before or during adolescence, while others only after adolescence, a further and final exploratory objective of the present study was to compare these two groups on the psychological dimensions considered.

In carrying out this study, we have taken into account that it is rare that adults with ABDL seek psychological or any other help [8,9,14,16]. Therefore, it is impossible to define the incidence of this phenomenon in the Italian population or refer to specific facilities or centres. For this reason, participants have been recruited by means of the main Italian ABDL online communities’ websites and thanks to the help of the Chair of the Italian AB Nursery Association. Indeed, adult-baby nurseries are the only place where an adult with ABDL can carry out childish activities and satisfy their own desire to be a baby.

## 2. Materials and Methods

### 2.1. Sample

Recruitment and testing conformed with the local Ethics Committee requirements and the Declaration of Helsinki. Participants joined the study on a voluntary basis. Before taking part in the research, participants were asked to read and approve an informed consent document. Participants were informed about privacy, the use of data, the lack of payment for participation, and the possibility of interrupting their participation in the study at any time, without any personal or legal repercussion. Complete anonymity was guaranteed to all participants. To be eligible for the study, participants had to be more than 18 years of age and to participate in at least one of the Italian ABDL online communities. The final sample was composed of 38 participants, 36 males and two females. The age of the participants ranged from 18 to 72 years (*M* = 34.95; *SD* = 12.25) and they were from different regions of Italy. The majority of the sample came from northern Italy. About 50% of the sample had a level of education of Secondary School, 77% of the sample did not have a current relationship, and 64.8% of the sample had a job. The main demographic characteristics are reported in Table 1.

### 2.2. Procedures 

The research procedure was implemented in a digital format and it was implemented using an online module. The module was organized into two sections, preceded by informed consent. The first section was created to collect the basic demographic information and anamnestic phenomena related to ABDL. The second section was designed to collect information about the psychological, medical, and personal history of the participants and to get a global evaluation of the participant’s psychological well-being. In particular, in the second section participants were administered all the scales included in the Cognitive Behavioural Assessment 2.0 battery [27], the Adult Parental Acceptance–Rejection Questionnaire [28], and the Personality Questionnaire Assessment [29]. To contact the wider range of participants from the online community, the Chair of the Italian AB Nursery Association was asked to share the link to take part in the study on the main websites and online social communities dedicated to the Italian ABDL adults or linked to the official website of the AB Nursery: www.abdl.forumcommunity.net; the Italian section of the international website www.abkingdom.com; and the Italian community of Facebook “Adult Baby d’Italia e del mondo”. A brief description of the general aims of the study was given with the link. Participants could participate in the study by following the link. The administration of the protocol started after they accepted the informed consent. Starting from October 18th, 2018, a link to the online module was presented. The entire procedure required about 90 minutes for completion.

### 2.3. Measures

#### 2.3.1. Ad Hoc Questionnaire 

An ad hoc questionnaire was developed to collect the basic demographic information (e.g., date of birth, gender, employment, civil status, educational qualification, and family structure). This questionnaire was also finalized to obtain information about the age of appearance of ABDL fantasies and possible concomitant events (“In the year of appearance of ABDL interests and/or fantasies, can you remember some special events that could have had an impact on that appearance”) and about the influence imprinted by special circumstances, mood states, and/or physical conditions on the desire to act ABDL (“According to you, are your ABDL behaviours linked to special events; circumstances; mood states, and/or physical conditions?”).

#### 2.3.2. Cognitive Behavioural Assessment 2.0 

To obtain a broad-spectrum measurement of psychological well-being, all participants were administered the Cognitive Behavioural Assessment 2.0 [27] battery compounded by seven tests.

##### State-Trait Anxiety Inventory—X Form

The State-Trait Anxiety Inventory—X1 Form and X2 Form [30] are composed of 20 items that indicate the presence of anxiety, tension, and discomfort, or the lack of these moods. The evaluation of mood provides for a 4-point Likert-type response format (from “not at all” to “very much”). The X1 Form evaluates the presence of anxiety state; it is an environmental anxious state. The X2 Form evaluates the presence of anxiety traits, which are personality dimensions that cause an increase in anxiety states in both anxious and not anxious circumstances. The battery also includes a short version of STAI-X1 (10 items) [27], which allows for an evaluation of the increase or the decrease of anxiety state while the subject is completing the protocol and it allows us to obtain an accuracy index of the battery. In the present study, the alphas for each score are adequate (αs > 0.955). 

##### Autobiographical Folder—Psychological Anamnesis 

The Autobiographical folder—psychological anamnesis [27] is composed of 56 items, with multiple choice and open-ended answers, to collect anamnestic information. This the part of test is meant to obtain information about the personal history, education, attitudes, and main relationship of the participant. Moreover, this section of C.B.A. 2.0 [27] evaluates some important psychological and clinical manifestations which are not included in other sections of the battery.

##### Eysenck Personality Questionnaire

The Eysenck Personality Questionnaire Short Form (EPQ-R) [27,31] was finalized to evaluate personality traits. The questionnaire is composed of 48 items subdivided into four scales: extroversion-introversion (E), emotional instability (N), social maladjustment (P) and control scale (L). Subjects must assess whether the content of the items describes itself, through dichotomous answers (yes–no). The significance of the EPQ-R is due to the ability of the questionnaire to investigate personality traits that represent a risk or protect factors of illness or disorder. In the present study, the alphas for each score are adequate (αs > 0.630).

##### Psychophysiological Questionnaire

The Psychophysiological Questionnaire Short Form (QPF-R) [32] is composed of 30 items that investigate the main psychophysiological reactions and disorders. Responses are collected on a 4-point Likert-type scale. Psychophysiological reactions reveal the interactions between somatic and psychological components, caused and reinforced by psychosocial stressors. The final score offers the possibility to know the propensity of individuals to minimize or to exaggerate the health state. In the present study, the alphas for each score are adequate (αs = 0.933).

##### Fear Inventory

The Fear Inventory Short Form (IP-R) [27] is the Italian short form of the Survey Schedule [33,34]. It is composed of 58 items that list some trigger situations connected with fear moods. The inventory is subdivided into five scales that, except for the first, investigate phobic reactions that are more frequently identified in clinical practice. Subjects should indicate the intensity of fear evoked by each item through a 5-point Likert-type scale, from “not at all” to “very much”. The final score offers an index of the nature and intensity of a subject’s fears. In the present study, the alphas for each score are adequate (αs = 0.957).

##### D Questionnaire

The D Questionnaire (QD) [27] evaluates depressive manifestations, as well as subclinical forms of depression. It is composed of 21 items that investigate the primary manifestations of depressive symptomatology, such as a depressive mood, sleeping disorder, self-devaluation. Subjects should indicate which of the statements matches with their own life. A high score is an index of depressive conditions, however, is not necessarily clinically relevant. In the present study, the alphas for each score are adequate (α = 0.936).

##### Maudsley Obsessive-Compulsive Questionnaire

The Maudsley Obsessive-Compulsive Questionnaire Short Form (MOCQ-R) [27,35] investigates obsessive-compulsive symptoms. The MOCQ-R is composed of 21 items, with “yes” or “no” answers. The questionnaire is subdivided into three scales: “Checking”, investigates the need to repeatedly verify everything; “Cleaning” explore the presence of obsessions and ritualistic behaviours concerned with hygiene, neatness, and fear of infections; “Doubt-Rumination”, concerns intrusive thoughts about things of irrelevant importance. In the present study, the alphas for each score are adequate (αs = 0.867). 

#### 2.3.3. Adult Parental Acceptance–Rejection Questionnaire

The short forms of Adult *Parental Acceptance–Rejection Questionnaire*, mother and father version (PARQ) [24,28] were administered. Based on these questionnaires, there is a theory known as IPAR Theory, which attempts to predict and explain the antecedents and the main consequences of perceptions of parental acceptance and rejection, empirically and independently of the cultural environment [26]. Both PARQ versions are composed of 24 items divided into four scales: warmth/affect; hostility/aggression; indifference/neglect; undifferentiated rejection. Subjects should indicate, on a 4-point Likert-type scale (from “almost always true” to “almost never true”), whether statements of each item describe the relationship both with their mother and father, when they were 7 to 14 years old. Total scores range from 24 (maximum acceptance perceived) to 96 (maximum rejection perceived). In the present study, the alphas for each score are adequate (αs > 0.762).

#### 2.3.4. Personality Assessment Questionnaire

To evaluate psychological adjustment, the Personality Assessment Questionnaire short form (PAQ) [24,29] was used. PAQ is a self-report questionnaire consisting of 42 items, which investigates seven personality dimensions: hostility-aggressiveness; dependence; negative self-esteem; inadequacy; lack of emotional response; and a negative world view. Subjects should think about personal feelings, ideals or desires, indicating their own degree of agreement on a 4-point Likert-type scale, from (4) “almost always true” through (1) “almost never true.” It is considered as a total score. A low total score indicates a balanced psychological adjustment. On the other hand, a high total score indicates psychological maladjustment. In the present study, the alphas for each score are adequate (αs > 0.735).

## 3. Data Analysis

To describe the demographic characteristics, the diffusion and the distribution of the ABDL phenomenon in the Italian community, the main descriptive analysis of considered variables was conducted. 

To investigate if the adults with ABDL showed more negative recollections of parental acceptance–rejection (both maternal and paternal) and a more negative psychological adjustment, separate one-sample z-tests were used to compare the mean scores of the ABDL sample and normative scores related to the Italian population. For each scale, normative data were taken from the scale handbook or the validation paper. To control the familywise type I error, the *p*-values were also corrected by using the False Discovery Rate method (FDR) [35].

Finally, to investigate if the age of appearance of ABDL fantasies was associated with the scores on the considered variables, the total sample was divided in two groups according to the age of appearance of ABDL fantasies. The first group (G1) contained the adults who reported the appearance of ABDL fantasies before 12 years; whereas the second group (G2) contained the adults who reported the appearance of ABDL fantasies after 12 years. The two groups were compared by means of one-way ANOVAs that considered the group as a between factor, and the considered psychological dimensions as dependent variables. To control the familywise type I error, the *p*-values were also corrected by using the False Discovery Rate method (FDR) [36].

All statistical analysis was conducted by means of IBM SPSS statistics 21 software (SPSS Inc, Chicago, IL, USA), and an alpha value of 0.05 was considered.

## 4. Results

Preliminary descriptive analyses were executed to investigate missing values and variable distributions. Univariate distributions of the observed variables were examined for normality [37]. These analyses showed no missing values or normality problem.

A descriptive analysis showed that the appearance of ABDL fantasies ranged from 3 to 30 years and that the average age was 11 years (*M* = 11.70; *SD* = 7.11). The first behaviours appeared in an age range from 5 to 35 years with an average age of 19 years (*DS* = 7.74); 55.3% of the sample placed the appearance of ABDL fantasies in childhood (0 to 11 years), the rest of the sample (44.7%) placed this appearance in adolescence or adulthood (older than 12 years). About 50% of the sample claimed that the first appearance of ABDL fantasies was linked to an event that influenced it: 15.8% indicated a physiological event (enuresis or issue of sphincter control) and 31.6% a psychological event (i.e., loss of a parent in childhood, lack of attention by parents, physical or psychological abuse, humiliating experiences). Moreover, 44.7% declared that, in general, ABDL behaviours are influenced by special circumstances, mood states, and/or physical conditions. Among these, the most frequent causes referred by participants (23.7%) is the avoidance and/or decrease of a negative mood state (i.e., frustration, sadness, stress, solitude, or need of protection). 

Other factors linked to ABDL behaviours concerned traumatic events (7.9%), incontinence (5.3%), and other factors, including sexual arousal and the satisfaction of a need (5.3%). With regard to anamnestic data, the results show that 57.9% of the sample did not have a sexual partner and/or limited their own sexual life to masturbation; 42.1% of the sample declared to be affected, or have been affected, by a loss of control of urine and/or stools; 57.9% of the sample declared to not have psychological issues at the time of the research; whereas, 36.8% of the sample affirmed having psychological distress (see Table 1). 

The comparison between the sample and the normative scores (see Table 2) showed that the ABDL adults had higher levels of perceived rejection for both maternal, *z* = 3.759, *p* < 0.001, and paternal relationships, *z* = 2.336, *p* = 0.009; higher scores on the state and trait anxiety dimensions, *z* = 1.872, *p* = 0.031 and *z* = 2.396, *p* = 0.008, respectively; and higher levels on both social dis-adjustment, *z* = 3.871, *p* < 0.001 and depression, *z* = 3.275, *p* < 0.001. No other significant differences were observed. The FDR correction confirmed the significance of *p*-values with the only exception being the state anxiety scale (STAI-X1).

The results of the ANOVA analysis are shown in Table 3. The data showed that adults who declared the genesis of ABDL fantasies before adolescence (G1) tended to show a lower psychological adjustment, a lower extroversion, a lower positive self-image, higher levels of trait anxiety, higher levels of emotional instability, higher levels of depressive and obsessive-compulsive manifestations, and a higher tendency to respond in a social desirability way, when compared with adults who collocated the genesis of ABDL fantasies after adolescence (G2). The FDR correction confirmed the significance of *p*-values with the only exception being the E scale.

## 5. Discussion

The first purpose of the study was to explore the main characteristics of the Italian ABDL population, the possible sources of ABDL phenomena and the main functions of ABDL behaviours. The data confirmed that, in the Italian population, ABDL phenomena involved males more than females. Participants came from different Italian regions, especially from Northern Italy. Italian ABDL persons seemed to have difficulties in their relationships. Most of the sample did not have a current relationship and, from the analysis of anamnestic responses, about half of the sample had not had a sexual partner and had limited their own sexual life to masturbation. No specific difficulties in the working area were observed. 

The first ABDL fantasies appeared mainly in the pre-adolescent ages (approximately 11 years), whereas the first ABDL behaviours appeared in adult ages (19 years). These characteristics are similar to the demographic characteristics observed by Hawkinson and Zamboni [15]. The average age of the appearance of ABDL fantasies was 11 years for males and 12 years for females. On the other hand, the age of appearance of ABDL behaviours was lower in the American ABDL sample than in the Italian ABDL sample (about 15 years). Half of the Italian ABDL sample associated the appearance of ABDL fantasies with a concomitant event. 

Among the referred events, enuresis was the most frequently reported. This association has already been described in the literature [9,11] and it was found also in Zamboni’s [15] study, where he assumed that the ABDL behaviours allow ABDL persons to face the discomfort derived from the loss of sphincterial functions, and to help them to accept their own physical condition. In our study, out of 38 participants, seven had directly linked the genesis of ABDL fantasies to infantile enuresis. Regarding the anamnestic data, the results show that about half of the sample declared to suffer, or to have suffered from enuresis. However, given the correlational design of this study, it is not possible to interpret the directionality of this association. Further consideration and analysis will be needed to clarify the nature of this association. Another important aspect highlighted from the literature review is the presence of different motivations underlying ABDL behaviours. For some people, ABDL behaviour could be of a sexual nature, for others, ABDL behaviours could be with the aim to satisfy the wish to be a baby, to re-live childhood, or to reduce a state of emotional tension and assume the function of a coping strategy. In fact, even though ABDL phenomena are considered an expression of atypical sexuality, it is not clear if the majority of ABDL interests are based on sexual motivations [15]. 

As assumed by Hawkinson and Zamboni [12], the presence of two different subgroups is possible. In the first subgroup, ABDL phenomena have a sexual nature, and in the second subgroup there is mainly infantile regression. Another point of view about the nature of ABDL phenomena is that ABDL behaviours play different types of functions, so the pursuit of pleasure is not, necessarily, the main function for all ABDL persons. This aspect seems to also be present in the Italian ABDL sample. 

The analysis of demographic characteristics showed two results that support the hypothesis that ABDL phenomenon is finalized to satisfy paraphilic components. The first component is the average age of ABDL fantasy appearance, at about 11 years old. This is usually the period in which there is the appearance of sexual instinct and consequentially, there is the manifestation of the first fantasies that can trigger sexual arousal [38]. Considering the sexual orientation development of the male, in this period it is highly probable for the sexualization of non-sexual triggers with a lack of appropriate external influences and the consolidation of paraphilic or atypical sexual orientation [39,40]. 

The second component, closely linked to the first one, is the higher involvement of males in the ABDL phenomena, in line with studies that have shown a male prevalence in paraphilic interests [41]. Nevertheless, only two subjects of the sample had directly linked ABDL fantasies to sexual arousal. This is consistent with the literature evidence about ABDL phenomena. In fact, even though ABDL phenomena are considered an expression of atypical sexuality, it is not clear if the grade of ABDL interests are based on sexual motivations [12]. 

In line with the literature regarding ABDL [3,8,10], some adults of the sample linked the genesis of ABDL fantasies to childhood abuse and trauma. Probably in these circumstances, the ABDL behaviour is firstly finalized to relive and to change their own childhood and the pursuit of sexual pleasure, which, if present, could be considered immaterial, compared to this function. Concerning ABDL as a dysfunctional coping strategy [10,11,12], an important finding of the present study concerns the presence of anxious traits, which predispose reactions when there is an increase of anxiety in front of triggers [30]. 

Furthermore, anxious traits determine a baseline emotional level, which is mostly negative [42]. The data also showed that the premature appearance of ABDL fantasies was associated with psychological distress. Moreover, about half of the participants believed that the ABDL phenomena were influenced by negative moods, such as sadness, frustration, and stress, or were motivated by the need for protection and safety, or the desire to escape from the real world. In the Italian ABDL sample, we found disseminated depressive traits. So, it is possible to hypothesize that, for Italian ABDL who participate in the online community, the ABDL phenomena could represent a coping strategy based on avoiding a negative mood state [43] through the pursuit of positive sensations generated by the regression to childhood and infantile behaviours. 

The decrease of discomfort and sense of relief linked to ABDL phenomena could be consolidated with the relation between ABDL interests and the avoidance of negative mood states, increasing the probability that ABDL individuals use ABDL phenomena to face stressful situations and other negative emotions [15]. Further consideration and analysis will be needed to clarify the possible functions of ABDL behaviours and to stabilize if it is more appropriate to talk about a behavioural continuum or two different subgroups in which there are different motivations for the appearance of ABDL interests and behaviours.

A second purpose of the study was to investigate the association between ABDL phenomena and the childhood relationships with caregivers [1,9,11,12,22]. In particular, we investigated if ABDL adults showed more negative recollections of parental acceptance–rejection and more negative psychological adjustment in general, compared with the general Italian population. Our data showed that some subjects of the sample had directly linked the genesis of ABDL interests to factors, including separation from parents, abandonment, lack of affection, or neglect. Moreover, the evaluation of recollections of parental rejection during childhood showed the presence of maternal and paternal relationships characterized by a lack of love, and the presence of hostility/aggression and undifferentiated rejection. 

It is possible to hypothesize the presence of a link between ABDL phenomena and the perception of parental reject during childhood, as experienced by Italian ABDL online members. Despite the fact that the results did not confirm the hypothesis of the presence of a more negative psychological adjustment in an Italian ABDL online sample, the presence of depressive and anxious traits could suggest psychological discomfort that did not express with the dimensions investigated by PARQ but through the tendency to a depressive mood state and generalized anxiety state. Moreover, some of the aspects that emerged are directly linked with childhood rejection. 

The scientific evidence shows that the individuals who feel rejected during childhood had a series of difficulties leading to interpersonal rejection. One of these difficulties concerns the creation of intimate relationships, based on sharing and the communication of thoughts and intimate feelings [25,28,44]. These persons would like intimacy; however, the tendency to consistently interpret events with distorted mental representations, resulting from rejection and the fear of other rejections, could reflect an underlying fear of intimacy, thus obstructing the creation of a meaningful relationship [25,26,45,46]. The lack of a steady relationship for about 77% of the sample could be a reminder of this fear of intimacy and of the need to defend themselves from other rejects. These difficulties also influence the sexual behaviour. 

In these circumstances, it was observed that sexual life was often confined to masturbation [47]. It is likely that this preference, also observed in the ABDL sample, was linked to a willingness to avoid the fear of intimacy with a sexual partner and this offered a valid alternative to reach sexual gratification [48]. 

Lastly, cognitive distortion resulting from the perception of rejection promotes the development of hypersensitivity for interpersonal rejection, real or perceived [38,48,49]. Analysing the fearful nature referred by ABDL subjects in IP-R, most of the sample has social fears, such as the fear of being judged or rejected. It is likely that the social fear reactions showed by the ABDL sample could be linked to this sensitivity. It is possible to hypothesize that Italian ABDL members of the online communities have a compromising of interpersonal development. The difficulty shown by the Italian ABDL sample in the creation and maintenance of sexual and/or affective relationships seems to confirm this hypothesis. Based on this interpersonal impairment, there could be an experience of rejection from parental relationships during childhood. Thus, ABDL phenomena could be another expression of parental rejection. The nature of this link needs to be studied in further research. 

Another purpose of the study was to exclude any underlying psychopathological condition causing ABDL phenomena. In particular, some scholars [7,9,11] hypothesized that the ABDL phenomenon was a manifestation of obsessive-compulsive disorder. This hypothesis was not confirmed for the Italian ABDL sample. The data showed that in the Italian ABDL sample, in general, relevant psychological issues were not present, with the exception of depressive manifestations. It is possible that ABDL is a distinct phenomenon, not caused by another primary psychological disorder; however, this aspect needs to be clarified in further research. 

Finally, considering the extreme variability of the age of appearance of the first ABDL fantasies and the subdivisions of the sample into two groups consisting in those with the genesis of ABDL fantasies in the pre-adolescent phase (0–12 years) and those with the appearance of ABDL fantasies in the post adolescent or adult age (older than 12 years), we decided to compare these two groups in all the psychological dimensions investigated. 

The data show that those who affirmed that the first ABDL fantasies appeared in childhood tended to have more psychological maladjustment, emotional instability, obsessive-compulsive behaviours, and negative moods, such as anxiety or depressive manifestations, than those who collocated the appearance of their first ABDL fantasies at an adult age. For the ABDL persons of the first group, the ABDL interests could reflect a rooted psychological discomfort and ABDL phenomena could be an expression of this psychopathological core, such as the other psychopathological dimensions measured. This due to the major impact of events associated with the genesis of ABDL fantasies and experienced during childhood on the future psychological health of the individual. 

Instead, the ABDL persons of the second group could experience the ABDL interests as a pleasing game and these activities do not reflect an underlying psychological discomfort. In fact, considering the presence of depressive and anxious traits in the Italian ABDL sample, the ABDL practices could be a way to decrease daily stress. Further research is needed to investigate if there could be other relevant events, for example, traumatic experiences from an early age and experiences connected with a perception of parental rejection, causing the genesis of a psychological discomfort that expresses itself in ABDL phenomena. In the Italian ABDL sample, we confirmed the lack of distress directly linked to ABDL phenomena generally found in literature [14]. Only two subjects of the sample affirm in the Autobiographical Folder to have sought the help of psychology for their own ABDL fantasies. 

## 6. Limitations

The first limitation concerns the reduced sample size and the research procedure adopted. Regarding the first limitation, a large number of ABDL people are not openly available in the nation. Due to this lack, the online procedure was preferred. Despite the limitations of the procedures caused by an online investigation, the online procedure was the only way to obtain a sample as representative as possible. Moreover, considering the delicacy of these phenomena, some people might have been hesitant to expose themselves directly. The absence of contact with psychological professionals makes the recruitment of ABDL persons difficult. Thanks to online methods, it was possible to reach a large number of people in order to obtain information about the diffusion of ABDL phenomena at the national level. Another limitation concerns the presence of only two females in the ABDL sample. A major involvement of males was attempted due to the same trend having been demonstrated in the literature [4]. Nevertheless, the presence of only two female subjects does not make it possible to deepen the differences of gender in ABDL phenomena. It could be interesting to investigate this aspect in future research.

## 7. Conclusions

This study was conceived with the aim to explore ABDL phenomena in an Italian ABDL online sample. Specifically, we wanted to obtain information about the demographic characteristics of Italian ABDL members from online communities and to find more information about the origins and primary functions of ABDL behaviours. We hypothesized that ABDL phenomena were associated with experiences of rejection experienced during childhood in parental relationships. We also hypothesized that this kind of relationship could be associated with a more negative adjustment in the Italian ABDL online sample. Considering Italian members of the ABDL online community, it is possible to affirm that the first ABDL fantasies appeared at about 11 years old, while the first behaviours appeared at about 19 years old. 

The appearance of ABDL fantasies seems to be caused mainly by certain factors, both physiological and psychological. Physiological factors concern sphincteric control troubles, while psychological factors concern aspects, such as childhood abuse and premature parent loss. ABDL behaviours could have different functions, including sexual gratifications or strategies to cope with negative mood states. Moreover, anxious and depressive traits were present in the Italian ABDL sample, coherently with the link between ABDL phenomena and negative mood states, as shown in the literature [10,11,12,22]. 

It is hypothesized that ABDL phenomena are derived by a parental relationship connoted by the rejection perception. Thus, ABDL could be one of the manifestations of the experience of rejection during childhood. This partially confirms the hypothesis of psychological maladjustment being linked to parental rejection, which is manifested through difficulties in interpersonal areas. Moreover, a negative psychological maladjustment is observed when the ABDL fantasies appeared during childhood (from 0 to 12 years). The age effect is interesting and should be studied in future research, as well as for clinic implications. 

In fact, it is possible that when ABDL fantasies appear during childhood there are other factors, with the perception of parental rejection, that determine a psychopathologic core. ABDL phenomena could be a symptomatic manifestation of that psychopathologic core. In this case, the contact with the online community could be linked to other factors, such as seeking the replacement of parental figures or a desire to re-live and change their childhood. 

Instead, for those who develop ABDL fantasies in adolescence or at an adult age, ABDL phenomena could represent pleasing and relaxing activities. Considering the disseminated presence of anxious and depressive traits, the use of online communities could represent a pleasing activity to decrease daily stress. Even in the Italian sample, as in the American one, this condition does not seem to be a cause of psychological distress; however, it is connected to collateral distress. The small sample size cannot allow a generalization of results for all ABDL people but could be indicative of stabilizing a trend concerning the Italian online ABDL community. 

In fact, it is possible that ABDL persons who use online communities are different from those who act out ABDL fantasies solitarily. Even though the limitations, including the small sample size and lack of a control group, the present study provides a starting point for future insights to increase the knowledge of ABDL phenomena. Increasing knowledge about ABDL is most important for clinicians and for adequate instrumentation for working with ABDL people. This is an important aspect considering that ABDL persons seem to be interested in understanding the nature of their ABDL fantasies and they could require psychological counselling to help them understand the origins and nature of ABDL interests.

## Figures and Tables

**Table 1:** Demographic characteristics and descriptive statistics. Adult Baby-Diaper Lovers (ABDL).

Variables	%
Residence	
Northern Italy	65.8
Central Italy	18.4
Southern Italy	7.9
Other	7.9
Level of Education	
Junior High School	13.9
Secondary School	50
University (3 years)	13.9
Master Degree	19.4
Post-Graduate degree	2.8
Civil State	
Unmarried	77.8
With relationship	5.6
Married	11.1
Divorced	5.6
Work	
Student	21.6
Employee	43.2
Self-Employed	21.6
Unemployed	10.8
Retired	2.7
Appearance of first ABDL fantasies	
Childhood	55.3
Adolescence or adult age	44.7
Triggers of ABDL fantasies	
Physiological	15.8
Psychological	31.6
ABDL behaviour influenced by triggers	
Yes	44.7
No	55.3
Triggers to ABDL behaviour	
Negative mood state	23.7
Traumatic events	7.9
Incontinence	5.3
Other	5.3

**Note.** Triggers of ABDL fantasies: events associated with the genesis of ABDL fantasies. Physiological: enuresis or issue of sphincter control; Psychological: i.e., the loss of a parent in childhood, a lack of attention by parents, physical or psychological abuse, humiliating experiences. ABDL behaviour influenced by triggers: behaviours of ABDL are influenced by particular circumstances, mood states, events, or physical conditions. Negative mood states: frustration, sadness, stress, solitude, and the need of protection. Other: sexual arousal; satisfaction of a need.

**Table 2:** One sample z-test results and descriptive statistics of a normative population.

Variables	Sample	Normative Population Mean		
	*M (SD)*	*Μ*	*Z*	*P*
PARQ Mother	46.9 (16.1)	36.95	3.759	<0.001 *
PARQ Father	47.5 (17.5)	40.78	2.336	0.009 *
PAQ	96.0 (24.22)	93.41	0.650	0.258
STAI-X1	44.6 (15.5)	39.83	1.872	0.031
STAI-X2	46.4 (16.2)	40.02	2.396	0.008 *
E	6.6 (3.6)	7.6	−1.690	0.954
N	5.8 (4.6)	4.6	1.587	0.056
P	3.3 (2.2)	1.9	3.871	<0.001 *
L	6.9 (3.1)	7.0	−0.196	0.578
QPF/R	45.1 (12.6)	43	1.014	0.155
IP-R	56.5 (32.4)	48.1	1.577	0.057
QD	6.4 (6.5)	2.9	3.275	<0.001 *
MOCQ-R	5.9 (5.8)	5.5	0.420	0.337

**Note.** PARQ Mother = Adult Parental Acceptance–Rejection Questionnaire, Mother Version, short form; PARQ Father = Adult Parental Acceptance–Rejection Questionnaire, Father Version, short form; PAQ = Personality Assessment Questionnaire, short form; STAI-X1 = State Trait Anxiety Inventory, X1 Forma; STAI-X2 = State-Trait Anxiety Inventory, X2 Form ; E = Estroversion-Introvation Scale; N = Emotional Instability Scales; P = Social Disadjustment Scales; L=Social Desirability; QPF/R = Psychophysiological Questionnaire; IP-R = Fear Inventory, short Form; QD = D Questionnaire; MOCQ-R = The Maudsley Obsessive-Compulsive Questionnaire, short Form. * *p-value* with FDR < 0.05.

**Table 3:** One-way ANOVA on the Major Study Variables as a function of the age of appearance of their first ABDL fantasies.

Variable	Sample		Total	Statistics			
	G1	G2					
	*M (SD)*	*M (SD)*	*M (SD)*	*F*	*df*	*P*	*η^2^_p_*
PARQ Mother	49.3 (15.1)	43.6 (17.2)	46.9 (16.1)	1.148	1.35	0.291	0.032
PARQ Father	50.8 (18.2)	43.2 (16.1)	47.5 (17.5)	1.761	1.35	0.193	0.048
PAQ	86.6 (21.1)	68 (21.1)	78.3 (22.8)	7.266	1.35	0.011 *	0.198
STAI-X1	47.1 (16.2)	41.3 (14.4)	44.6 (15.5)	1.292	1.35	0.263	0.036
STAI-X2	52.2 (15.2)	38.7 (14.5)	46.4 (16.2)	7.453	1.35	0.010 *	0.176
E	5.5 (3.5)	7.9 (3.4)	6.6 (3.6)	4.433	1.35	0.042	0.112
N	7.4 (4.4)	3.7 (4.2)	5.8 (4.6)	6.357	1.35	0.016 *	0.154
P	3.8 (2.5)	2.7 (1.8)	3.3 (2.2)	1.894	1.35	0.177	0.051
L	5.7 (2.6)	8.5 (3.0)	6.9 (3.1)	9.227	1.35	0.004 *	0.209
IP-R	63 (33.9)	47.3 (28.8)	56.5 (32.4)	2.351	1.35	0.134	0.063
QD	8.6 (6.7)	3.5 (5.0)	6.4 (6.5)	6.422	1.35	0.016 *	0.155
MOCQ-R	7.8 (5.9)	3.3 (4.7)	5.9 (5.8)	6.249	1.35	0.017 *	0.151

* *p-value* with FDR < 0.05.
